# A Comprehensive Review of Natural Products Against Allergic Rhinitis and Asthma: From Sensitization to Chronic Remodeling

**DOI:** 10.3390/ijms27073171

**Published:** 2026-03-31

**Authors:** Xuesong Zhang, Wenchu Zhou, Jie Zhang, Chenggang Liu

**Affiliations:** 1 School of Basic Medical Sciences, Heilongjiang University of Chinese Medicine, 24 Heping Road, Harbin 150040, China; zhangtaibai123456@163.com; 2 Graduate School, Heilongjiang University of Chinese Medicine, 24 Heping Road, Harbin 150040, China; rita_zhouwenchu@163.com (W.Z.); zhangjie24010@163.com (J.Z.)

**Keywords:** allergic rhinitis, allergic asthma, natural small molecules, chronopharmacology, sensitization, acute exacerbation, airway remodeling

## Abstract

Allergic rhinitis (AR) and allergic asthma are chronic airway inflammatory diseases characterized by three phases: sensitization, acute exacerbation, and chronic remodeling. While conventional antiallergic drugs provide symptomatic relief, they often face limitations including drug resistance, side effects, and inability to reverse chronic airway remodeling. Natural products have emerged as promising therapeutic alternatives due to their multi-target effects and safety profiles. This review systematically summarizes natural small molecules targeting distinct pathological mechanisms across the three phases of AR and asthma, introducing a chronopharmacological perspective for stage-specific therapeutic strategies. During sensitization, flavonoids (quercetin, luteolin, apigenin, baicalin) and polyphenols (curcumin, resveratrol) target the epithelial–dendritic cell axis by suppressing alarmin release and blocking dendritic cell maturation. In acute exacerbation, flavonoids (hispidulin, quercetin) and isoquinoline alkaloids (coptisine) exhibit rapid intervention through mast cell stabilization and neurogenic inflammation suppression. In chronic remodeling, stilbenes (resveratrol) and flavones (baicalin, baicalein) reverse established structural changes through TGF-β1/Smad, PTEN/PI3K/AKT, and PDGF-BB/PDGFR-β pathways. Mapping natural compounds to specific disease stages provides a molecular basis for precision medicine approaches.

## 1. Introduction

**Figure 1:**
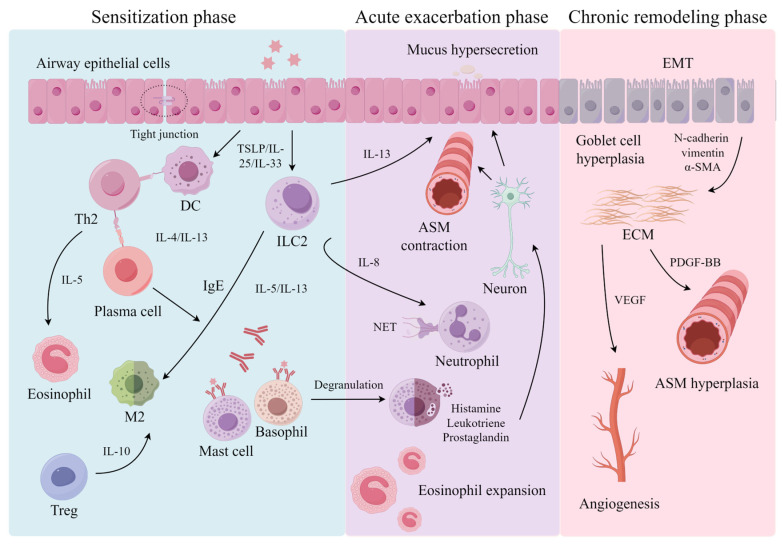
Schematic overview of the three phases of allergic airway inflammation. The sensitization phase (blue) begins with allergen exposure, triggering epithelial alarmins (TSLP, IL-33, IL-25) that activate DCs and ILC2s. This drives Th2 differentiation, IgE production, eosinophil infiltration, M2 macrophage polarization, and Treg suppression, establishing a self-amplifying inflammatory loop. The acute exacerbation phase (purple) involves mast cell degranulation via IgE cross-linking, releasing histamine and leukotrienes that cause smooth muscle contraction and vascular leakage. This is followed by neutrophil NETosis, mucus hypersecretion via IL-13/STAT6 signaling, and neurogenic inflammation, creating persistent feedback. The chronic remodeling phase (pink) features epithelial–mesenchymal transition (EMT) with cadherin switching, ASM hypertrophy driven by PDGF-BB, angiogenesis via VEGF, excessive ECM deposition with cross-linking, goblet cell hyperplasia with aberrant MUC5AC glycosylation, and neuronal proliferation, collectively leading to irreversible airway obstruction and hyperresponsiveness.

Allergic rhinitis (AR) is an IgE-mediated inflammation of the nasal mucosa, marked by congestion, rhinorrhea, sneezing, and itching [1], while allergic asthma involves reversible airway obstruction and high IgE levels [2]. Both conditions, which often occur together [3,4,5], can be classified by duration and severity according to ARIA and GINA guidelines [3,6]. Their global prevalence is rising, which significantly exacerbates the overall disease burden [7,8,9]. They share similar pathogenic mechanisms, including sensitization, acute exacerbation, and chronic remodeling phases [10]. In the sensitization phase, allergens activate dendritic cells, promoting naive T cells to differentiate into Th2 cells, leading to IgE production and mast cell activation [11]. In the acute exacerbation phase, allergen–IgE complexes trigger mast cell degranulation, releasing substances that cause immediate nasal symptoms [12]. In the chronic remodeling phase, interleukin (IL)-1/IL-13 activate type 2 innate lymphoid cells (ILC2s) and recruit eosinophils, leading to persistent inflammation and tissue remodeling in AR [13]. Asthma follows a similar pattern: Th2 polarization and IgE prime-dependent mast cell in the sensitization phase [14]; mast cell and eosinophil degranulation cause bronchoconstriction in the acute exacerbation phase [15]; and sustained ILC2 activation and eosinophilic infiltration lead to airway smooth muscle (ASM) hypertrophy and extracellular matrix (ECM) deposition in the chronic remodeling phase [16]. Both diseases share similar pathogenic processes (Figure 1).

Due to the limitations of traditional antiallergic drugs, such as drug resistance, side effects, and inability to reverse chronic remodeling, natural products are gaining attention for their multi-target effects, safety, and regulatory properties [17]. Research shows that natural ingredients, like flavonoids, alkaloids, and terpenoids, can modulate immune functions and inhibit inflammation [18]. However, current reviews often take a “list-style” approach, focusing on a single disease or compounds, which oversimplifies the complex nature of allergic diseases. Werner et al. reviewed natural small-molecule compounds that inhibit mast cell or basophil activation but treated these effects as uniform across all disease stages, not distinguishing early IgE-mediated processes and later tissue remodeling [19]. While recent reviews have detailed the antiallergic actions of natural small molecules in specific diseases, they overlook varying disease stages. Reviews on herbal medicines for AR cite the inhibition of NF-κB, MAPK, and STAT3 as general mechanisms, but they do no differentiate between early alarmin release and later mucous gland hyperplasia [20]. Similarly, a handbook chapter on asthma and chronic obstructive pulmonary disease (COPD) categorizes natural products by chemical families and describes their anti-inflammatory effects without distinguishing between acute bronchoconstriction and chronic ASM proliferation [21]. Liu et al.’s review categorizes antiallergic bioactives by chemical class but overlooks their varying efficacy during different allergy phases [22]. Disease-specific reviews, like those on atopic dermatitis, often focus on downstream signaling pathways without considering phase-specific differences of these pathways [23].

Recent studies have examined antiallergic compounds such as ellagitannins and tetramethylpyrazine, but tend to treat their effects as uniform, ignoring phase-specific activities [24,25]. A review on flavonoid delivery highlights improved bioavailability but ignores the impact of release timing on therapeutic outcomes [26]. Reviews on nutrient polyphenols discuss antiallergic effects without considering timing [27]. A ginseng review focuses on static anti-inflammatory and antioxidant effects in the nasal mucosa [28]. A ginger mini-review notes mast cell histamine suppression but misses its stronger impact on Th2 cytokine production in the acute phase than chronic tissue remodeling [29]. These studies generally overlook disease stages when discussing the antiallergic properties of natural small molecules.

This review addresses this gap by introducing the chronopharmacology, detailing their actions during sensitization, acute exacerbation, and chronic remodeling in AR and asthma. This perspective explains the varying effectiveness of natural products in research models over time and provides a molecular basis for “preventive treatment” by targeting early sensitization. By mapping natural compounds to disease stages, it guides stage-specific therapies, allowing precise intervention in disease progression and supporting the development of targeted antiallergic drugs.

This narrative review was conducted following a structured literature search strategy to ensure transparency and reproducibility. We systematically searched PubMed, Embase, Web of Science, and Scopus databases from January 2010 to February 2026 using the following keyword combinations: (“natural products” OR “flavonoids” OR “alkaloids” OR “terpenoids” OR “polyphenols”) AND (“AR” OR “allergic asthma”) AND (“sensitization” OR “acute exacerbation” OR “chronic remodeling” OR “airway inflammation”). Inclusion criteria were: (i) peer-reviewed original articles and reviews published in English; (ii) studies clearly defining disease phases (sensitization, acute exacerbation, or chronic remodeling); (iii) natural small molecules with documented antiallergic mechanisms. Exclusion criteria included: (i) studies lacking clear phase-specific definitions; (ii) non-peer-reviewed sources; (iii) studies focusing solely on crude extracts without identification of active compounds. This approach ensures that the phase-specific framework presented reflects the most robust available evidence while acknowledging the limitations of current research.

## 2. Sensitization Phase

Upon exposure to allergens, epithelial cells release alarmins, such as thymic stromal lymphopoietin (TSLP), IL-33, IL-25, that activate dendritic cells (DCs), which then drive naive T cells to a Th2 phenotype through the STAT6-GATA3 pathway [30,31,32,33]. This triggers an immune response where Th2 cells secrete IL-4 and IL-13, aiding B cells in producing allergen-specific IgE [34,35]. Alarmins activate ILC2s to release IL-5 and IL-13, leading to eosinophil maturation and recruitment [36,37]. Th2 cytokines shift macrophages to an M2 phenotype, inducing reactive oxygen species (ROS) generation [38]. IgE binds to mast cells and basophils, sensitizing them. Epithelial cells produce IL-5 and eotaxin (CCL11), which extend eosinophil lifespan and recruit them via CCR3. This results in eosinophil infiltration and degranulation in the airway/nasal mucosa [39,40]. Alarmins and Th2 cytokines (IL-4, IL-13) disrupt the epithelial barrier by affecting tight junction [41,42] and suppress regulatory T cell (Treg) function by inhibiting Foxp3 and IL-10 [43]. Early-life gut microbiota and its metabolites, especially short-chain fatty acids (SCFAs), are crucial for stable Foxp3^+^ Treg populations. Imbalance here can lead to uncontrolled Th2 responses [44,45]. This triggers a self-amplifying inflammatory loop involving epithelial alarmin release, DC-mediated Th2 priming, IgE production, mast cell/eosinophil activation, M2 polarization, barrier dysfunction, mucus metaplasia, and impaired Treg function. This loop contributes to the sensitization phase of AR and asthma, leading to acute exacerbations upon allergen exposure. Research shows that natural small molecules can exert anti-allergic effects by intervening in the pathological context mentioned above (Table 1).

### 2.1. Natural Small Molecules Block Allergen Sensitization by Modulating the Epithelial–DCs Axis

Recent studies indicate that natural small molecules can suppress the release of alarm cytokines (IL-33, TSLP, IL-25), blocking DC activation and reducing their maturation and antigen-presentation capacity. In experimental models, compounds like quercetin and an ephedrine-derived alkaloid from *Ephedra sinica* demonstrate the ability to alleviate allergic symptoms by targeting the epithelial–DC axis. In ovalbumin (OVA)-induced mouse models, quercetin reduces IL-33, IL-25, and TSLP secretion and downregulates CD80/CD86 on DCs, inhibiting their maturation and migration [46,47]. Baicalin attenuates type 2 immune responses in a mouse allergic asthma model through inhibiting the production of TSLP [48]. Similarly, in an OVA-challenged allergic asthma model, the amide alkaloid suppresses epithelial proteinase-activated receptor-2 signaling (IL-33, TSLP) and reduces DC maturation markers (CD80, CD86, and MHC class II), preventing their migration [49]. Curcumin reduces lung inflammation and influences DC morphology and function by activating the Wnt/β-catenin signaling in mouse model of asthma [50]. Systematic reviews confirm these polyphenol compounds suppress alarm cytokines (IL-33, TSLP, IL-25) and downregulate MHC class II and DC co-stimulatory molecules (CD83, CD80, CD86) [27,51], effectively alleviating AR and asthma symptoms. These preclinical findings provide mechanistic insights, though clinical validation of these specific epithelial–DC axis modulations in human allergic disease remains to be fully established.

### 2.2. Natural Small Molecules Attenuate the Expansion of Memory Th2 Cells and the Massive Secretion of IL-4 and IL-13

Natural small molecules can effectively slow disease progression in AR and allergic asthma by targeting key pathways during early sensitization. In an OVA-sensitized asthma mouse model, curcumin disrupts Th2 polarization by blocking the Notch 1 receptor, reducing NICD1 levels, and decreasing GATA3 activity, IL-4, and IL-13 production, which prevents memory Th2 cell formation [52]. Apigenin inhibits p-STAT6 in OVA-challenged mice, reducing GATA3 expression and Th2 cytokines (IL-4, IL-5, IL-13), thereby blocking Th2 polarization and memory responses [53]. Luteolin ameliorated inflammation and Th1/Th2 imbalance via regulating the TLR4/NF-κB pathway in AR rats [54]. Quercetin attenuates OVA-induced rubbing and sneezing by balancing the Th1/Th2 cells ratio in AR mice [55]. These compounds attenuate the expansion of memory Th2 cells and the massive secretion of IL-4 and IL-13 during the sensitization phase.

### 2.3. Natural Small Molecules Inhibit Allergic Sensitization via Modulating IgE–FcεRI Interaction

Evidence shows that natural small molecules have strong antiallergic activities by targeting key steps in the sensitization phase, such as IgE regulation and mast cell/basophil modulation. In the OVA-induced mouse model of allergic asthma, baicalin ameliorates asthma-associated pathological alterations by reducing serum IgE levels and repressing type 2 immune responses [48]. Resveratrol inhibits IL-33-mediated mast cell activation by targeting the MK2/3-PI3K/Akt axis [56]. Hispidulin and osthole mitigate allergic sensitization in asthma by downregulating FcεRI expression on mast cells, blocking IgE–FcεRI interactions, and inhibiting the Syk/PI3K/STAT6 signaling pathway [57,58]. Hispidulin also activates the Nrf2/HO-1 antioxidant pathway to reduce oxidative stress, while osthole lowers serum IgE levels and inhibits Th2 cytokine production [57,58]. These actions decrease mast cell and basophil sensitization, reducing airway inflammation and hyperresponsiveness in OVA-induced allergic asthma models. Tanshinone IIA reduced mast cells in the nasal mucosa and lowered the levels of histamine, OVA-IgE, OVA-IgG1, TNF-α, IL-4, and IL-5, thereby alleviating nasal symptoms in an OVA-induced mouse model of AR; it also inhibited 30% of histamine release in Compound 48/80 (C48/80)-induced HMC-1 cells [59]. All these compounds target core immune pathways to interfere with allergic processes.

### 2.4. Natural Small Molecules Attenuate M2 Polarization by Targeting IL-4/IL-13/STAT6 and Downstream Signaling Pathways

Natural small molecules can disrupt the IL-4/IL-13/STAT6 signaling pathway to inhibit M2 polarization. This reduce the expression of tissue repair markers like Arg-1 and Fizz-1 and blocks TGF-β1 precursor formation. Bakuchicin, for example, prevents STAT6 acetylation and affects the TRAF4-AKT/mTOR Myc signaling, reducing M2 polarization in an OVA-induced mouse model [60]. Similarly, cynaropicrin suppresses allergic lung inflammation through M2 polarization by inhibiting the galectin-3 pathway [61]. In an OVA-induced asthma model, luteolin inhibits the IL-33/ST2-GSK3β axis, blocking STAT6 activation and reducing M2 marker expression, while boosting IL-10 production and activating the PI3K/Akt pathway to shift M2 macrophages to an anti-inflammatory state [62]. These suggest that natural small molecules like bakuchicin, resveratrol, and luteolin can reprogram macrophage polarization by targeting molecular pathways such as STAT6, TRAF4-AKT/mTOR-Myc, and galectin-3, offering insights into treating allergic airway inflammation.

### 2.5. Natural Small Molecules Inhibit the Rapid Recruitment and Activation of Eosinophils

Eosinophil recruitment is crucial in acute AR and asthma flare-ups. In a mouse model of HDM-induced asthma, lonicerin significantly reduced airway hyperresponsiveness and eosinophil infiltration by inhibiting the Src/EGFR signaling pathway, lowering the levels of IL-4, IL-5, IL-13, and eotaxin-1/2/3, suggesting its potential as a treatment for allergic asthma [63]. Similarly, in an OVA-induced mouse model of AR, galangin showed strong antiallergic effects by modulating the PI3K/Akt pathway, reducing the production of eotaxin, IL-4, IL-6, IL-13, and IgE, and decreasing eosinophil infiltration, thereby alleviating nasal symptoms [64]. Oroxylin A reduced airway hyperresponsiveness in OVA-challenged BALB/c mice by inhibiting eosinophil infiltration in bronchoalveolar lavage fluid (BALF) [65]. Recent studies highlight the therapeutic potential of natural small molecules in acute asthma and AR by reducing eosinophil infiltration with downregulating eotaxins and cytokines, as well as inhibting the Src/EGFR signaling pathway and PI3K-AKT signaling pathway.

### 2.6. Natural Small Molecules Restore Epithelial Barrier Integrity and Suppress Immune Activation by Modulating the TSLP/IL-33-Tight Junction Axis

A growing number of studies have shown that natural small molecules can alleviate allergic responses by regulating the interaction between the epithelial barrier and immune cells during the sensitization phase. Alpha-linolenic acid upregulated occludin and zonula occludens-1 (ZO-1) expression by activating the JAK2/STAT3 signaling pathway, improving nasal inflammation and epithelial barrier damage, and strengthening epithelial barrier function [17].

### 2.7. Natural Small Molecules Ameliorate Allergic Inflammation by Modulating the Treg/Th17 Balance

Recent studies indicate that natural small molecules like quercetin, luteolin, and curcumin have antiallergic effects by modulating T cell function. Quercetin attenuated OVA-induced rubbing and sneezing by increasing IL-10 and Foxp3 levels, balancing Treg/Th17 cells via NF-κB pathway inhibition, thereby attenuating AR [55]. Luteolin restored Treg/Th17 balance to ameliorate AR in a mouse model, decreasing IL-10 and Foxp3 levels [66]. Curcumin suppressed IL-4, IL-8, and TNF-α and increased IL-10 production through the inhibition of NF-κB signaling pathway [67].

### 2.8. Natural Small Molecules Regulate Allergic Inflammation by Modulating the Gut Microbiota–SCFA–Treg Axis

Oral resveratrol boosts gut microbes *Akkermansia muciniphila* and *Bacteroides acidifaciens*, raising SCFAs like acetate and butyrate. This inhibits HDAC9, enhancing Foxp3 expression and IL-10 production, restoring the Treg–IL-10 axis and reducing eosinophilic infiltration, and IL-5, IL-13, and MUC5AC transcription [68]. In OVA-induced asthmatic mice, tetrahydrocurcumin, a curcumin metabolite, alters the gut microbiota by lowering the Firmicutes/Bacteroidetes ratio and increasing *Akkermansia* and *Bacteroides*, thereby raising butyrate production. This activates G protein-coupled receptors (GPR)43 and GPR109A, upregulating Foxp3 and IL-10 to improve pulmonary Treg function, while also suppressing epithelial alarmins TSLP, IL-33, and IL-25, as well as restoring tight junction proteins E-cadherin and ZO-1 to prevent barrier leakage [69].

**Table 1:** Natural small molecules targeting distinct pathological mechanisms during the sensitization phase of AR and asthma.

Disease Context	Name	Chemical Type	Research Model	Research System	Year	Reference
Epithelial alarmin release and DC activation	Quercetin	Flavonoid	OVA-induced mouse model	Mouse model	2023 2016	[46] [47]
	Baicalin	Flavonoid	OVA-induced allergic asthma mouse model	Mouse model	2025	[48]
	Amide alkaloid	Alkaloid	OVA-challenged allergic asthma model	Mouse model	2022	[49]
	Curcumin	Polyphenol	Mouse model of asthma	Mouse model	2017	[50]
Th2 priming	Curcumin	Polyphenol	OVA-sensitized asthma mouse model	Mouse model	2014	[52]
	Apigenin	Flavonoid	OVA-challenged mice	Mouse model	2020	[53]
	Luteolin	Flavonoid	AR rats	Mouse model	2021	[54]
	Quercetin	Flavonoid	AR mice	Mouse model	2023	[55]
IgE production, mast cell/basophil activation	Baicalin	Flavonoid	OVA-induced mouse model of allergic asthma	Mouse model	2025	[48]
	Resveratrol	Polyphenol	IL-33-mediated mast cell activation model	In vitro system	2019	[56]
	Hispidulin	Flavonoid	OVA-induced allergic asthma models	Mouse model	2024	[57]
	Osthole	Coumarin	LL-37-induced rosacea mice; C48/80, substance P, LL-37 or (R)-ZINC-3573-stimulated LAD2, skin mast cells, and mouse peritoneal cells	Mouse model and in vitro system	2020	[58]
	Tanshinone IIA	Diterpenoid	OVA-induced mouse model of AR; C48/80-induced HMC-1 cells	Mouse model and in vitro system	2022	[59]
M2 polarization	Bakuchicin	Coumarin	OVA-induced mouse model	Mouse model	2024	[60]
	Cynaropicrin	Sesquiterpene lactone	Allergic lung inflammation model	Mouse model	2018	[61]
	Luteolin	Flavonoid	OVA-induced asthma model	Mouse model	2025	[62]
Eosinophil activation and recruitment	Lonicerin	Flavonoid	HDM-induced asthma mouse model	Mouse model	2022	[63]
	Galangin	Flavonoid	OVA-induced mouse model of AR	Mouse model	2024	[64]
	Oroxylin A	Flavonoid	OVA-challenged BALB/c mice	Mouse model	2016	[65]
Barrier dysfunction	Alpha-linolenic acid	Fatty acid	Nasal inflammation model	Mouse model	2025	[17]
Impaired Treg function	Quercetin	Flavonoid	OVA-induced AR mice	Mouse model	2023	[55]
	Luteolin	Flavonoid	AR mouse model	Mouse model	2023	[66]
	Curcumin	Polyphenol	Patient with perennial AR with chronic episodic allergic asthma	Human study	2022	[67]
Gut microbiota–SCFA–Treg axis	Resveratrol	Polyphenol	HDM-induced asthma model	Mouse model	2022	[68]
	Tetrahydrocurcumin	Polyphenol	OVA-induced asthmatic mice	Mouse model	2021	[69]

## 3. Acute Exacerbation Phase

During acute exacerbations of AR and asthma, the pathological processes intensify in a layered and time-sequential manner. When allergens bind with IgE–FcεRI receptors on mast cells, it triggers degranulation through the Lyn/Syk-PLCγ-IP_3_-calcium signaling pathway, releasing mediators like histamine, tryptase, and TNF-α [70,71,72]. This is followed by the production of prostaglandin D_2_ and leukotrienes, which increase vascular permeability and cause smooth muscle contraction and nasal swelling [73,74]. Tryptase promotes IL-8/CXCL8 production [75]. Neutrophils migrate to the site, forming neutrophil extracellular traps (NETs) and releasing matrix-degrading enzymes like matrix metallopeptidase (MMP)-9 and neutrophil elastase (NE), worsening tissue damage and marking acute exacerbation [76]. IL-13 from Th2 cells and ILC2s increase MUC5AC/MUC5B transcription via STAT6 [77]. In addition, acetylcholine and the parasympathetic nervous system stimulate mucus hypersecretion [78]. Neurogenic inflammation, through histamine and PGE2, activates TRPV1/TRPA1 receptors, creating a nasobronchial reflex [79]. Neuropeptides enhance bronchial smooth muscle contraction and mucus secretion, while airway stimulation worsens nasal inflammation, creating a persistent feedback loop [80]. Overall, this temporal network is initiated by mast cell degranulation, followed by smooth muscle contraction and mucus hypersecretion, eosinophil expansion, neutrophilic infiltration, and neurogenic inflammation, driving the acute exacerbation phase. Emerging evidence demonstrates that natural small molecules exert anti-allergic effects through intervention in the aforementioned pathological mechanisms (Table 2).

### 3.1. Natural Small Molecules Regulate Mast Cell and Basophil Degranulation Through Multi-Target Intervention

Recent studies show that natural small molecules can inhibit mast cell and basophil activation, reducing degranulation and tissue damage during acute exacerbations. In OVA-induced mouse asthma models and RBL-2H3 cells, hispidulin inhibits the FcεRI signaling pathway and activate the Nrf2/HO-1 antioxidant pathway, decreasing calcium influx and histamine and β-hexosaminidase release, thus reducing airway inflammation [57]. Additionally, quercetin attenuates MRGPRX2-mediated mast cell degranulation via the MyD88/IKK/NF-κB and PI3K/AKT/Rac1/Cdc42 pathway in CLM-1-knockdown LAD2 cells stimulated by C48/80 [81]. Coptisine inhibits mast cell degranulation by suppressing the PI3K/Akt signaling pathway based on an in vitro model (RBL-2H3 cells sensitized with DNP-IgE/HSA) and an in vivo model (OVA-induced AR mice) [82]. These findings reveal that natural small molecules precisely regulate mast cell and basophil degranulation by targeting signaling pathways like FcεRI, MRGPRX2, NF-κB, PI3K/Akt, and Nrf2/HO-1, providing key targets for new antiallergic treatments.

### 3.2. Natural Small Molecules Inhibit Airway Smooth Muscle Contraction and Mucus Hypersecretion

Excessive airway smooth muscle (ASM) contraction and mucus hypersecretion, key features of acute asthma attacks, involve a complex contraction network. Research shows that natural small molecules can modulate ASM contraction, offering potential treatments for the acute exacerbation phase. In guinea pig tracheal ring models, quercetin has shown concentration-dependent inhibition of contractions caused by KCl, acetylcholine, tetraethylammonium, and histamine [83]. In TGF-β1-treated ASM and OVA-induced mouse asthmatic models, curcumin nanoparticles (CUR-NPs) inhibit ASM migration by suppressing the TGF-β1/p-STAT3/CTGF signaling pathway [84]. Additionally, during acute exacerbations, resveratrol downregulates the mCLCA3/hCLCA1 signaling pathway to reduce MUC5AC expression in an OVA-challenged murine asthma model [85]. These findings demonstrate that natural small molecules can regulate ASM regulation contraction and mucus hypersecretion by TGF-β1/p-STAT3/CTGF and mCLCA3/hCLCA1.

### 3.3. Natural Small Molecules Suppress Eosinophil and ILC2 Expansion by Targeting the Epithelial-ILC2

Recent studies show that natural small molecules can exert antiallergic effects by modulating the expansion of eosinophils and ILC2 cells. Calycosin reduced eosinophil number in BALF of OVA-exposed asthmatic mice [86]. Bilirubin suppressed ILC2 proliferation through downregulation of ERK phosphorylation and GATA3 expression in IL-33-induced mouse airway inflammatory model [87]. In IL-33-induced mouse models, ILC2 activation represents a common pathway that could be targeted by natural compounds. These findings suggest that modulation of eosinophil and ILC2 populations represents a viable therapeutic strategy, though evidence from AR and asthma models remains limited.

### 3.4. Natural Small Molecules Reduce Early Neutrophil Infiltration and Neurogenic Inflammation

Recent studies show that natural small molecules have inhibitory effects on neutrophil infiltration. Quercetin decreased neutrophil infiltration in several asthma models [88]. In addition, in a TDI-induced rat model of AR, quercetin (≥25 mg/kg, for 5 days) significantly reduced the levels of substance P (SP), calcitonin gene-related peptide (CGRP), and NGF in the nasal lavage fluid, accompanied by an alleviation of nasal irritation symptoms [89]. These findings highlight the key role of quercetin in the acute exacerbation phase of allergic airway diseases.

**Table 2:** Natural small molecules targeting distinct pathological mechanisms during the acute exacerbation phase of AR and asthma.

Disease Context	Name	Chemical Type	Research Model	Research System	Year	Reference
Mast cell and basophil degranulation	Hispidulin	Flavonoid	OVA-induced mouse asthma models; RBL-2H3 cells	Mouse model and in vitro system	2018	[57]
	Quercetin	Flavonoid	CLM-1-knockdown LAD2 cells stimulated by C48/80	In vitro system	2024	[81]
	Coptisine	Alkaloid	RBL-2H3 cells sensitized with DNP-IgE/HSA; OVA-induced AR mice	Mouse model and in vitro system	2018	[82]
Airway smooth muscle contraction	Quercetin	Flavonoid	Guinea pig tracheal ring models	Mouse model	2016	[83]
	Curcumin nanoparticles (CUR-NPs)	Polyphenol	TGF-β1-treated ASM; OVA-induced mouse asthmatic models	Mouse model and in vitro system	2024	[84]
Mucus hypersecretion	Resveratrol	Polyphenol	OVA-challenged murine asthma model	Mouse model	2016	[85]
Eosinophil and ILC2 expansion	Calycosin	Flavonoid	OVA-exposed asthmatic mice	Mouse model	2024	[86]
	Bilirubin	Tetrapyrrole	IL-33-induced mouse airway inflammatory model	Mouse model	2022	[87]
Neutrophil infiltration	Quercetin	Flavonoid	Several asthma models	Mouse model and in vitro system	2020	[88]
Neurogenic inflammation	Quercetin	Flavonoid	TDI-induced rat model of AR	Mouse model	2016	[89]

## 4. Chronic Remodeling Phase

AR and asthma, despite their different symptoms, share similar underlying mechanisms, involving chronic airway remodeling. This process is driven by epithelial–mesenchymal transition (EMT), ASM hypertrophy, angiogenesis, excessive extracellular matrix (ECM) deposition, goblet cell hyperplasia, and neuronal remodeling [90,91,92]. In a Th2-type inflammatory environment, airway epithelial cells undergo EMT, marked by decreased E-cadherin and increased mesenchymal markers like N-cadherin, vimentin, and α-SMA [91], leading to epithelial barrier disruption and cell migration into the airway wall. These cells then secrete fibrotic proteins like collagen I/III, causing subepithelial fibrosis, which underlies airway narrowing and airway hyperresponsiveness [91]. Abnormal activation of platelet-derived growth factor (PDGF-BB) and its receptor PDGFR-β causes ASM cells to shift from a contractile to a synthetic phenotype, leading to excessive proliferation and the secretion of collagen I/III and fibronectin, thickening the airway wall. These cells also release angiogenic factors such as VEGF, promoting angiogenesis and contributing to airway remodeling [91]. Allergen-induced VEGF-A/VEGFR2 signaling pathway [93] and Ang-1/Tie2 imbalance increase vascular permeability, worsening inflammatory cell infiltration, matrix cross-linking, and collagen deposition [94,95]. This results in turbinate hypertrophy and bronchial congestion, diminishing the effectiveness of anticholinergic drugs [96]. During remodeling, ECM shows excessive deposition, with continuous synthesis of Type I, III, and V collagens and a relative decrease in elastin, reducing tissue compliance [97,98,99]. The upregulation of lysyl oxidase (LOX) facilitates irreversible collagen fiber cross-linking, worsening airway wall stiffness [100,101]. Despite increased MMP-9 activity, significant TIMP-1 upregulation causes an imbalance in matrix degradation, leading to net deposition [102]. Abnormal mucus secretion is crucial for airway hyperresponsiveness [103]. Allergen-induced stimulation causes Clara cells transdifferentiate into goblet cells, with continuous demethylation of the MUC5AC promoter [104,105]. The increased high-mannose-type MUC5AC proportion enhances mucus viscoelasticity, forming difficult to clear mucus plugs that obstruct the airway and promote airway hyperresponsiveness through physical obstruction [106,107]. In addition, neuronal remodeling further reinforces airway hyperresponsiveness by increasing cholinergic/sensory nerve fiber density and branching due to chronic inflammation [108,109]. This nerve proliferation lowers the threshold for airway hyperresponsiveness, causing overreaction to stimuli like cold air and smoke, leading to persistent neuronal hypersensitivity [110,111]. In AR and asthma, this chronic remodeling forms a complex network of reinforcing processes. Early intervention targeting EMT and inflammatory pathways may stop airway remodeling progression, improving symptoms and patient outcomes. Natural small molecules have been shown to exert anti-allergic effects through intervention in the pathological processes described here (Table 3).

### 4.1. Natural Small Molecules Can Inhibit Chronic-Phase EMT by Blocking the Synergistic TGF-β1/IL-4/IL-13 Signaling

The TGF-β1/Smad signaling pathway and the PI3K/AKT signaling pathway represent two of the most consistently validated mechanisms in AR and asthma and all three phages, with reproducible findings across multiple laboratories and model systems [112]. However, the PDGF-BB/PDGFR-β pathway involvement in ASM hyperplasia is well-established in rodent models but requires further confirmation in human studies [113]. In chronic inflammation, persistent TGF-β1 signaling and Th2 cytokines (IL-4/IL-13) drive EMT, marked by decreased E-cadherin and increased N-cadherin, Vimentin, and α-SMA, leading to epithelial barrier destruction and subepithelial fibrosis [114,115,116]. Resveratrol inhibits the TGF-β1/Smad signaling pathway, reducing the expression of EMT transcription factors such as Snail and Slug, restoring E-cadherin, and decreasing vimentin and α-SMA in an OVA-induced asthma model [117]. It also upregulates PTEN, inhibiting EMT via the PI3K/AKT pathway, thus alleviating airway remodeling and epithelial barrier damage [118]. Quercetin blocks Twist1 in the TGF-β1 signaling pathway, increasing E-cadherin and reducing mesenchymal markers like vimentin and α-SMA in an OVA-induced asthma model [119,120]. EGCG enhances PTEN expression, inhibiting the PI3K/AKT signaling pathway and TGF-β1-induced EMT, reducing TGF-β1 production in airway epithelial cells, decreasing Smad phosphorylation, and restoring E-cadherin expression, while lowering vimentin and α-SMA expression in an OVA-induced asthmatic mouse model [121]. Curcumin ameliorates inflammation and fibrosis by affecting MAPK signaling pathway and downregulating MMP-9 and α-SMA expression in OVA-induced asthmatic mouse model [122]. Naringenin attenuates OVA-induced airway remodeling in chronic asthmatic mice by inhibiting peribronchial fibrosis and downregulating α-SMA [123]. These compounds help to alleviate airway inflammation and prevent remodeling and fibrosis, making them promising candidates for treating the chronic remodeling phase of AR and asthma by restoring epithelial cell function and inhibiting mesenchymal transition.

### 4.2. Natural Small Molecules Precisely Target ASM Hyperplasia to Inhibit Chronic Airway Remodeling

During the chronic remodeling phase, ASM hyperplasia is mainly driven by the PDGF-BB/PDGFR-β signaling pathway, with IL-33/ST2 promoting ASM cells to transform into synthetic phenotype, increasing collagen I/III secretion. This results in airway wall thickening and disordered ASM cell arrangement [124]. Resveratrol upregulates PTEN, reversing PDGF-BB-induced Akt phosphorylation, inhibiting ASM proliferation [118]. Baicalin reduces remodeling-related cytokines such as IL-13, VEGF, TGF-β1, MMP-9, and tissue inhibitor of metalloproteinase 1 (TIMP1) in an OVA-induced asthmatic mice model [125]. In chronic asthma mouse models, resveratrol taken orally reduces subepithelial collagen deposition by inhibiting the HMGB1/TLR4/NF-κB pathway [126,127]. Curcumin attenuates airway remodeling by altering ASM thickening in a murine model of chronic asthma [128]. Meanwhile, baicalein attenuates OVA-induced airway inflammation and airway remodeling by reducing collagen deposition and ASM thickness [129].

### 4.3. Natural Small Molecules Block Angiogenesis and Vascular Remodeling During the Chronic Remodeling Phase by Inhibiting VEGF Signaling or Modulating the Angiopoietin/Tie2 System

Quercetin effectively reduces nasal symptoms and inhibits angiogenic factors such as VEGF and basic fibroblast growth factor (bFGF) in OVA-induced murine AR model [130]. Meanwhile, baicalein specifically targets VEGFA and epidermal growth factor receptor (EGFR) in allergic asthma models to reduce airway angiogenesis [129]. Luteolin inhibits Gas6-induced angiogenesis by blocking growth arrest-specific gene 6 (Gas6)/Axl signaling pathway, and Gas6 is significantly upregulated in mice with allergic airway disease [131,132]. Overall, these natural small molecules target angiogenic factors to prevent vascular changes in AR and asthma.

### 4.4. Natural Small Molecules Curb ECM Over-Deposition and Cross-Linking During Remodeling by Modulating the MAPK/AKT Signaling Pathway and Inhibiting NF-κB Signaling Pathway

Baicalin downregulates IL-1β-stimulated ECM production, migration, and contraction by inhibiting p-MAPK, p-Akt, p-p50, p-p65, and p-IκBα in IL-1β-stimulated nasal fibroblasts [133]. In an OVA-sensitized and -challenged mouse chronic asthma model, naringenin attenuated chronic inflammation by reducing collagen deposition [123]. Collectively, these natural small molecules ameliorate ECM over-deposition and cross-linking by downregulating MAPK/AKT signaling pathway and NF-κB signaling pathway.

### 4.5. Natural Small Molecules Reduce Goblet Cell Hyperplasia

Allergic asthma and rhinitis exhibit goblet cell hyperplasia, leading to mucus hypersecretion and changes in mucus rheology, causing mucus retention, airway obstruction, and impaired lung function. Glycyrrhizin has been confirmed to inhibit goblet cell hyperplasia and MUC5AC mRNA expression in mouse lung tissue induced by LPS or IL-4 through the inhibition of MUC5AC gene transcription [134]. Similarly, naringenin inhibits airway remodeling in a model of chronic asthma by reducing goblet cell hyperplasia [123]. Overall, glycyrrhizin and naringenin reduce goblet cell hyperplasia to alleviate chronic airway remodeling.

**Table 3:** Natural small molecules targeting distinct pathological mechanisms during the chronic remodeling phase of AR and asthma.

Disease Context	Name	Chemical Type	Research Model	Research System	Year	Reference
EMT	Resveratrol	Polyphenol	OVA-induced asthma mice	Mouse model	2017 2015	[117] [118]
	Quercetin	Flavonoid	TGF-β1-induced invasion in SW480 cells; OVA-induced asthmatic rats	Mouse model and in vitro system	2018 2023	[119] [120]
	EGCG	Polyphenol	OVA-induced asthmatic mice	Mouse model	2018	[121]
	Curcumin	Polyphenol	OVA-induced asthmatic mice	Mouse model	2023	[122]
	Naringenin	Flavonoid	OVA-sensitized and -challenged chronic asthma mice	Mouse model	2014	[123]
ASM Hyperplasia	Resveratrol	Polyphenol	OVA-induced asthmatic mice; Chronic allergic airway mice; OVA-induced asthma rats	Mouse model	2015 2011 2019	[118] [126] [127]
	Baicalin	Flavonoid	OVA-induced asthmatic mice	Mouse model	2023	[125]
	Curcumin	Polyphenol	OVA-induced chronic asthma mice	Mouse model	2014	[128]
	Baicalein	Flavonoid	OVA-induced airway inflammation and remodeling model	Mouse model	2024	[129]
Angiogenesis and vascular remodeling	Quercetin	Flavonoid	OVA-induced AR mice	Mouse model	2021	[130]
	Baicalein	Flavonoid	OVA-induced asthma mice	Mouse model	2024	[129]
	Luteolin	Flavonoid	Allergic airway disease mice	Mouse model	2017 2014	[131] [132]
ECM deposition	Baicalin	Flavonoid	IL-1β-stimulated nasal fibroblasts	In vitro system	2016	[133]
	Naringenin	Flavonoid	OVA-sensitized and -challenged chronic asthma mice	Mouse model	2014	[123]
Goblet cell hyperplasia	Glycyrrhizin	Triterpenoid saponin	LPS or IL-4 induced mouse lung tissue	Mouse model	2010	[134]
	Naringenin	Flavonoid	OVA-sensitized and -challenged chronic asthma mice	Mouse model	2014	[123]

## 5. Conclusions and Future Direction

Based on the evidence presented in this review, natural small molecules exhibit distinct therapeutic advantages across the three pathological phases of AR and asthma, with clear stage-specific patterns emerging. Unlike conventional pharmacotherapies—which typically target single molecules (e.g., glucocorticoids acting on glucocorticoid receptors, antihistamines blocking H1 receptors, leukotriene receptor antagonists targeting cysteinyl LTRs, or monoclonal antibodies such as omalizumab and dupilumab directed against IgE or IL-4Rα)—natural small molecules often exert multi-target effects by simultaneously modulating multiple signaling pathways and cell populations across both AR and asthma. This polypharmacological characteristic enables natural compounds to intervene at multiple stages of the disease continuum, potentially addressing aspects that single-target drugs cannot.

During the sensitization phase, flavonoids (quercetin, luteolin, apigenin, baicalin) and polyphenols (curcumin, resveratrol, EGCG) demonstrate the most robust preventive effects. These compounds predominantly target the epithelial–DC axis by suppressing alarmin release (TSLP, IL-33, IL-25) and blocking DC maturation. Notably, quercetin emerges as a particularly versatile agent in this phase, simultaneously inhibiting epithelial alarmins, balancing Th1/Th2 responses, and restoring Treg function through NF-κB pathway inhibition. The gut microbiota–SCFA–Treg axis modulation by resveratrol and tetrahydrocurcumin represents a novel preventive strategy, highlighting the importance of oral bioavailability and metabolic conversion for long-term immune tolerance.

During acute attacks, flavonoids (hispidulin, quercetin) and isoquinoline alkaloids (coptisine, berberine-related structures) exhibit the strongest direct-acting effects through immediate mast cell stabilization and neurogenic inflammation suppression. Hispidulin stands out by dual-targeting FcεRI signaling and Nrf2/HO-1 antioxidant pathways, effectively reducing calcium influx and degranulation. Remarkably, quercetin demonstrates unique neuroimmunomodulatory properties in this phase, significantly reducing Substance P, CGRP, and NGF levels in nasal lavage fluid, thereby attenuating neurogenic inflammation. This positions quercetin as a bridge compound connecting immune and neuronal mechanisms.

In the chronic remodeling phase, stilbenes (resveratrol, pterostilbene analogs) and flavones (baicalin, baicalein, oroxylin A) show superior efficacy in reversing established structural changes. Resveratrol demonstrates comprehensive anti-remodeling activity by inhibiting EMT via TGF-β1/Smad and PTEN/PI3K/AKT pathways, suppressing ASM hyperplasia through PDGF-BB/PDGFR-β blockade, and reducing angiogenesis. Baicalin and baicalein effectively target collagen deposition, ASM thickness, and vascular remodeling. The ability of these compounds to modulate multiple remodeling pathways simultaneously (EMT, ASM proliferation, angiogenesis, ECM deposition) suggests their potential as disease-modifying agents rather than merely symptomatic treatments. This review describes the canonical disease progression model; however, individual patient trajectories may vary, with some developing overlapping phases or bypassing certain phases entirely due to disease severity, comorbidities, or environmental triggers.

Effective implementation of stage-specific natural product therapy requires clear identification of transition points between disease phases. From sensitization to acute exacerbation, elevated serum IgE levels combined with increased nasal lavage tryptase and neuropeptides (Substance P, CGRP) may serve as early biomarkers signaling imminent clinical symptoms, indicating the need for rapid-acting mast cell stabilizers like hispidulin or coptisine. From acute to chronic remodeling, persistent elevation of TGF-β1, increased circulating EMT markers (decreased E-cadherin, increased vimentin), and imaging findings showing fixed airway obstruction (reduced FEV1/FVC ratio unresponsive to bronchodilators) suggest progression to the remodeling phase, necessitating intervention with anti-fibrotic agents such as resveratrol or baicalin. Additionally, MMP-9/TIMP-1 imbalance in induced sputum and increased circulating VEGF levels may indicate ongoing subepithelial fibrosis and angiogenesis. Incorporating these biomarkers into clinical decision-making algorithms would enable precise timing of natural product interventions, maximizing their therapeutic window and preventing irreversible structural damage.

Despite strong preclinical evidence, significant challenges remain in applying these findings to clinical practice. It is important to acknowledge that the majority of mechanistic studies cited in this review were conducted in experimental animal models (primarily OVA-induced murine models) or in vitro systems, which limits direct extrapolation to human clinical contexts. While these preclinical studies provide essential insights into molecular mechanisms and phase-specific targets, clinical validation remains limited for many of the discussed natural compounds. Therefore, the therapeutic advantages proposed in this review represent potential applications based on mechanistic plausibility and early-phase clinical observations, rather than established clinical efficacy across all discussed stages. Future studies must prioritize translational validation to bridge the gap between preclinical promise and clinical reality, particularly regarding bioavailability, dosing, and long-term safety in human subjects with allergic airway diseases. Future trials should, on the one hand, stratify patients by disease phase: Test flavonoids for prevention in steroid-naïve patients with early sensitization, quercetin or coptisine for rapid relief in acute exacerbations, and resveratrol or baicalin for modifying disease in patients with fixed airway obstruction. On the other hand, they should use biomarker-guided endpoints: Include phase-specific biomarkers like alarmin levels for sensitization, mast cell tryptase and neuropeptides for acute phases, and EMT markers, ASM mass, and ECM cross-linking for chronic remodeling, beyond just symptom scores.

While natural products are generally perceived as safer alternatives to synthetic drugs, several compounds discussed in this review warrant careful safety evaluation. Despite its broad antiallergic effects, quercetin may interact with cytochrome P450 enzymes (particularly CYP3A4 and CYP2C9), potentially affecting metabolism of co-administered drugs such as warfarin, cyclosporine, and certain chemotherapeutic agents. High doses have been associated with renal toxicity in rodent studies [135]. At high doses (>2.5 g/day in human studies), resveratrol has been associated with gastrointestinal distress, diarrhea, and potential interactions with anticoagulant medications (warfarin, clopidogrel) due to platelet aggregation inhibition [136]. The active component of licorice can cause pseudoaldosteronism with prolonged use (>2 weeks) due to 11β-hydroxysteroid dehydrogenase type 2 inhibition, leading to hypertension, hypokalemia, and edema [137]. Future clinical trials should incorporate comprehensive safety assessments, including dose-ranging toxicity studies, evaluation of compound–drug interactions, and long-term safety monitoring. The risk–benefit profile of natural products must be carefully weighed, particularly for chronic conditions requiring prolonged treatment.

Current evidence indicates that natural scaffolds need optimization to enhance bioavailability while retaining multi-target benefits. Quercetin glycosides like rutin have better stability but limited membrane permeability, whereas aglycones are quickly metabolized. Site-specific methylation or methoxylation could enhance metabolic stability and activity. Pterostilbene’s better oral bioavailability than resveratrol, due to less glucuronidation, highlights methylation of phenolic hydroxyls as a promising strategy. Curcumin phosphate esters or quercetin prodrugs could improve absorption and enable tissue-specific activation. The success of CUR-NPs in inhibiting ASM migration should be applied to other poorly soluble compounds, focusing on targeted delivery to nasal or lung tissues using ligand-conjugated nanoparticles. In addition, pharmacokinetic profiles often show short half-lives, necessitating multiple daily administrations that may compromise patient compliance. Dose translation from animal models to human equivalent doses remain problematic due to species differences in metabolism. Additionally, compound standardization poses regulatory challenges, as natural product compositions vary with plant source, extraction method, and geographic origin. Structural optimization strategies—such as methylation to enhance metabolic stability (e.g., pterostilbene versus resveratrol) or nanoparticle encapsulation (e.g., curcumin nanoparticles)—offer promising approaches to address these limitations while preserving the multi-target therapeutic advantages that distinguish natural products from single-target drugs.

While resveratrol and tetrahydrocurcumin modulate gut microbiota to enhance Treg function, the specific bacterial strains and metabolites responsible remain unidentified. Future research should focus on: (1) Isolating bacterial effectors by identifying metabolites from *Akkermansia muciniphila* or Bacteroides species that mediate antiallergic effects. (2) Developing synbiotic formulations by combining natural molecules with specific probiotics to boost SCFA production and Treg induction. (3) Assessing nasal microbiota to see if topical flavonoids can alter nasal bacteria and suppress harmful microbes.

## Data Availability

No new data were created or analyzed in this study. Data sharing is not applicable to this article.

## Abbreviations

AKT, protein kinase B; AR, allergic rhinitis; ASM, airway smooth muscle; BALF, bronchoalveolar lavage fluid; C48/80, Compound 48/80; DCs, dendritic cells; ECM, extracellular matrix; EMT, epithelial–mesenchymal transition; FcεRI, high-affinity IgE receptor; CGRP, calcitonin gene-related peptide; HDM, house dust mite; IgE, immunoglobulin E; IL, interleukin; ILC2s, type 2 innate lymphoid cells; LOX, lysyl oxidase; MAPK, mitogen-activated protein kinase; MMP, matrix metallopeptidase; MRGPRX2, Mas-related G protein-coupled receptor X2; NETs, neutrophil extracellular traps; NF-κB, nuclear factor kappa B; OVA, ovalbumin; PDGF, platelet-derived growth factor; PI3K, phosphoinositide 3-kinase; ROS, reactive oxygen species; SCFAs, short-chain fatty acids; STAT, signal transducer and activator of transcription; TGF, transforming growth factor; Th2, T helper 2; TIMP, tissue inhibitor of metalloproteinases; Treg, regulatory T cells; TSLP, thymic stromal lymphopoietin; VEGF, vascular endothelial growth factor.
